# An integrated online-to-offline model for HIV post-exposure prophylaxis (O2O-PEP) scale-up among men who have sex with men (MSM): Protocol for developing a pilot randomized controlled trial

**DOI:** 10.3389/fpubh.2022.1026137

**Published:** 2022-11-16

**Authors:** Qianqian Luo, Yongchuan Luo, Tianying Li, Tianyu Cui

**Affiliations:** ^1^School of Nursing, Binzhou Medical University, Yantai, China; ^2^Yantai Affiliated Hospital of Binzhou Medical University, Yantai, China

**Keywords:** online-to-offline model, post-exposure prophylaxis (PEP), men who have sex with men, HIV, protocol

## Abstract

**Background:**

HIV post-exposure prophylaxis (PEP) is an evidence-based biomedical HIV prevention strategy consisting of a 28-day course of highly active antiretroviral therapy after recent potential exposure to HIV. However, awareness and uptake of PEP among men who have sex with men (MSM) are very low. Innovative and effective methods are needed to support PEP implementation among MSM. This work reports a protocol to design and evaluate an online-to-offline-based delivery model for HIV PEP uptake (O2O-PEP) in Chinese MSM.

**Methods and analysis:**

This will be a two-phase study. In phase 1, we will develop an O2O-PEP model delivered through the WeChat mini-app (an app built into the WeChat platform). The O2O-PEP model initially includes four core components: a gamification-based education package for PEP, an online HIV risk assessment tool, a free online booking system for PEP initiation, and offline PEP prescription in the study hospitals. In phase 2, a two-arm pilot stratified randomized controlled trial comparing the O2O-PEP group with the standard care group will be designed to assess the feasibility, usability, and preliminary evidence of the efficacy of the O2O-PEP model in increasing PEP uptake among Chinese MSM. Model feasibility and usability will be further explored for broader model implementation.

**Discussion:**

The O2O-PEP model is one of the first interventions in China aiming to promote PEP initiation in Chinese MSM. Components in the O2O-PEP model could assist MSM in better understanding their HIV infection risk and increasing accessibility of PEP. Moreover, coupled with online and offline recruitment, the O2O-PEP model has great potential to reach and engage MSM who are not involved in care by traditional methods.

**Clinical trial registration:**

No. ChiCTR2200062538.

## Introduction

The HIV epidemic continues to be a major public health challenge among men who have sex with men (MSM) globally (1). Systematic reviews indicate an increasing HIV prevalence among MSM in China from 1.4% in 2001 to 8% in 2015 (2, 3). In larger, urban areas of China, MSM has been found to account for more than 50% of all newly diagnosed HIV infections (4). A wide range of comprehensive interventions has been adopted to resolve this challenge, with an emphasis on post-exposure prophylaxis (PEP) and pre-exposure prophylaxis (PrEP) (5, 6).

PEP is an evidence-based HIV prevention strategy in which a 28-day course of antiretroviral medication is administered to uninfected persons within 72 h (preferably within 24 h) after a recent possible exposure to HIV to minimize the risk of acquiring HIV. The effectiveness of the PEP in reducing HIV transmission has been reported in different studies (7, 8), and guidelines in different countries have been developed for PEP usage (9–12), including China (6). However, the overall uptake of PEP among MSM remains relatively low. Generally, only 4–6% of MSM who engaged in high-risk sexual behaviors (e.g., had unprotected anal intercourse with casual partners or substance use during anal sex in the past 6 months) reported having used PEP (13). Qualitative and quantitative studies reported multiple barriers to PEP uptake among MSM including low awareness of HIV infection risk, lack of PEP accessibility, and concerns about sexual risk compensation after PEP usage (14–16). Stigma against homosexuality and PEP usage further inhibits the effective delivery of PEP services for MSM (17). Therefore, innovative and effective approaches are needed to support PEP implementation in MSM.

Nearly universal mobile phone ownership provides MSM an opportunity to move away from traditional ways of meeting partners, such as bars, to seeking sexual partners through geosocial networking applications, and instant messaging apps. Integrated online-to-offline (O2O) models for HIV prevention are feasible and acceptable among MSM due to their help in identifying and engaging key populations, and seamlessly transitioning from online to offline services. O2O models in HIV service practice refer to the linkage between online service utilization and subsequent offline clinical service uptake and had been adopted in promoting the uptake of PrEP usage and HIV testing among MSM (18, 19). To date, China has relied heavily on traditional offline outreach models for PEP scale-up, despite the inherent challenges of engaging hard-to-reach MSM (20). This gap identifies a need for exploring O2O model implementation to promote PEP uptake in MSM.

Our study proposes to design and evaluate an O2O-based delivery model for HIV PEP uptake in Chinese MSM (O2O-PEP). The O2O-PEP model mainly comprises four components: a gamification-based PEP education package, an online HIV infection risk assessment tool, a free online booking system, and offline PEP prescription and related health services. We hypothesize that this O2O-PEP model could successfully bridge online outreach and offline PEP uptake and promote PEP uptake and continuation in MSM.

## Methods and materials

### The theoretical foundation for the O2O-PEP delivery model

**Figure 1:**
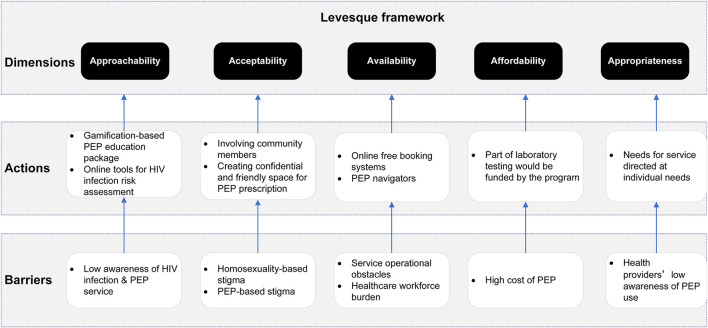
The conceptual framework for the postexposure prophylaxis (PEP) uptake intervention. The conceptual framework in our study is informed by the Levesque framework, including five domains of accessibility of health services: approachability, acceptability, availability, affordability, and appropriateness.

Figure 1 displays the study's conceptual framework. The O2O-PEP model is developed following the Levesque framework (21), which conceptualizes five dimensions of accessibility of health services: approachability, acceptability, availability, affordability, and appropriateness, with five corresponding abilities attributed to health users: the ability to perceive, ability to seek, ability to reach, ability to pay, and ability to engage. The Levesque framework has been widely used in intervention studies, including promoting HIV testing and PrEP usage (22).

### Development of the O2O-PEP model

**Figure 2:**
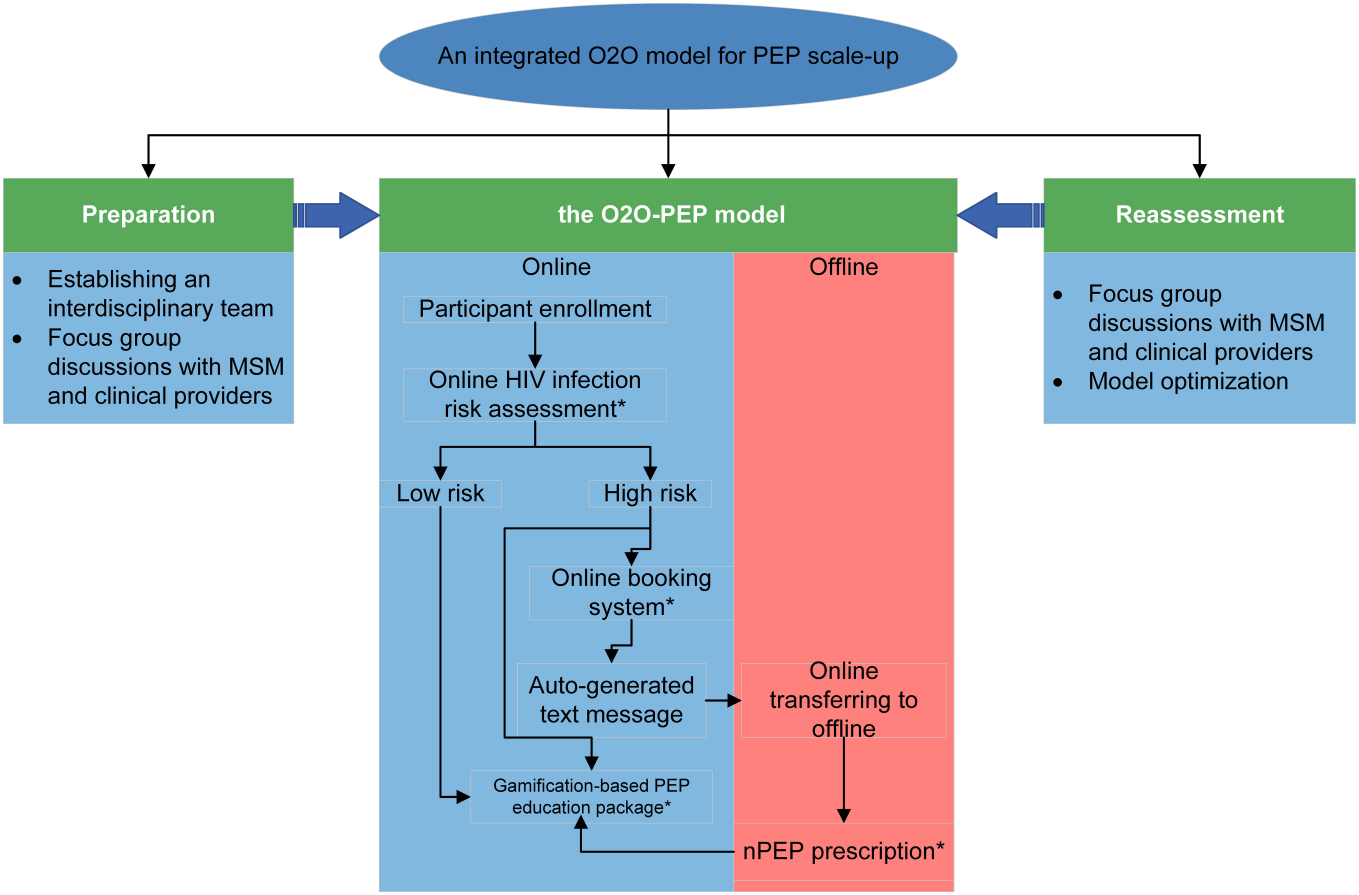
Overview of O2O-PEP model. O2O, Online to Offline, PEP, Post-Exposure Prophylaxis.

We followed the recommendations by Anand et al. (23) to design an integrated O2O model. The overview of the model construction is presented in Figure 2 and contains the following four steps:

#### Step 1. Establishing an integrated interdisciplinary team

Our interdisciplinary team consists of an online service team, an offline service team, and a technology team. The online service team includes HIV researchers and community-based organization (CBO) working staff, whose main task is to develop online PEP healthcare education packages and provide e-counseling support when needed. A doctor and a nurse in a hospital specializing in HIV management compose the offline service team, whose main task is to start a PEP regimen (the doctor) and PEP counseling for MSM (the nurse). The O2O-PEP model will be developed with VeiRui Technology (Beijing, China), a firm specializing in developing gamification-based software. The authors (QQL, TYC, and TYL) will be directly involved in creating requirements for the model.

#### Step 2. The first round of focus group discussions among Chinese MSM and healthcare providers

This step contains several FGDs with different populations before the model development: (1) MSM living in Qingdao, Beijing, and Jiangsu, China; (2) HIV-related service providers in CBOs; and (3) clinical health workers who are working in public health centers for HIV management (e.g., hospitals and centers for disease prevention and control). FGDs cover the following topics: HIV and PEP knowledge (only for MSM); current use of and access to WeChat and other mini-app technologies, and their potential role in promoting HIV prevention; opinions about the components of the model and what other functions should be included in this model; whether the model would be used after the construction; and how the model should be incorporated into routine HIV service sessions (only for healthcare providers). Online and offline in-person FGDs are both adopted; online FGDs use the cloud-based Tencent meeting, which provides an easy-to-use video conference that enables users to host or join meetings anytime anywhere. Each FGD lasts ~1 h and is conducted by 3 trained facilitators who are familiar with the study goals.

#### Step 3. Building version 1.0 of the O2O-PEP model

During this period, we partner with VeiRui Technology to build version 1.0 of the O2O-PEP model. Preliminary data from step 2 will be analyzed to build the model and applied through the WeChat mini-app. WeChat is an instant messaging and social media app developed by Tencent (Tencent Holdings Ltd., China), and it has been widely used in chronic disease management and HIV interventions (24, 25). The WeChat mini-app is an app built within the WeChat platform, which provides its users with an all-in-one experience. The O2O-PEP model initially consists of the following four main components.

#### An online HIV infection risk assessment tool

To identify assessment questions that could be used for recommending PEP usage, we will first review existing HIV infection risk assessment tools or variables positively associated with HIV infection, and then search PEP usage-related guidelines in different countries. The final assessment questions would include the time lag between sexual exposure and initial assessment (<72 h), and variables related to sexual exposures and sexual partners. According to users' responses to this tool, they will be divided into two groups: high risk of HIV infection vs. low risk of HIV infection.

#### Gamification-based education package for PEP

Gamification is a simulation technology using game design elements (e.g., challenges, points, and rewards) in non-game contexts. Advantages of gamification include (1) no time- and location-boundedness, as it provides low-risk learning, (2) less resource requirement as it offers contactless learning without a teacher, and (3) users' increased level of focus and attention as the unpredictability is embedded within the simulation (26, 27). Some studies have adopted gamification design in HIV interventions, such as reducing shame levels and risky sexual behaviors in MSM and increasing self-efficacy for HIV status disclosure in HIV-positive MSM (28, 29). The specific goals of the gamification-based education package for PEP in our study include (1) improving awareness and self-efficacy of PEP uptake, and (2) helping MSM understand the potential consequences of complying with or discontinuing PEP usage. When users involve in this gamification, they are always in situations where they must make decisions about PEP usage and corresponding consequences based on their choice, for instance, using or not using PEP, compliance with or discontinuation of PEP follow-up. Users' curiosity about “what will happen when they make different choices” is key to user engagement in the module. They are also exposed to valuable learning materials such as PEP usage timelines, laboratory testing, and caveats for PEP usage.

#### Free online booking system

Participants can click on the booking system link on the screen at any time during their immersion in the gamification to make online bookings anonymously and schedule appointments for their offline clinic visits. If participants schedule successfully, they will receive an auto-generated text message confirming the booking summary, which includes an e-ticket with booking details, a map to the clinic site, and a quick response (QR) code. The code is later scanned by the clinical service provider using a mobile app at check-in.

#### Offline PEP prescription and related health services

When MSM transfer from online to offline health services, healthcare providers should first confirm the participants' identity by scanning the QR code generated by online bookings for MSM. Then the doctor in the hospital starts the PEP regimen following the national PEP guideline (6), which includes laboratory testing and PEP prescription. The nurse in the same hospital is mainly responsible for PEP-related counseling, such as acute HIV infection symptoms, PEP-related side effects, risky behavior reduction, and PEP follow-ups.

#### Step 4. The second round of FGDs for model refinement and external usability test

After completion of version 1.0 of the O2O-PEP model, we will further conduct FGDs with MSM and healthcare workers who will be sent to the preliminary model to test the model and solicit opinions on the functionality of the mini-app. Glitches internal to the O2O-PEP model will be remedied during this period, including issues with log-ins, navigation, functionality, and para-data collection. These FGDs will be conducted by the same three facilitators in step 2.

After developing a polished version of the O2O-PEP model, we will conduct a 1-week, single-arm, pilot study among ten MSM aged 18 years or older to assess whether users could successfully navigate the model. MSM who participated in previous FGDs are encouraged to participate in this pilot test, as their feedback will provide useful insights to the research team. Upon completion of the pilot test, all participants will complete an online individual in-depth interview with study staff to provide feedback on functionality, bugs encountered, potential safety concerns, and experiences using the model. These ten participants would also complete the System Usability Scale (SUS) testing for the model usability assessment (30). SUS is a ten-item Likert Scale (0–10) giving an overall view of subjective assessment of the usability of a variety of products including websites and mobile phones, with a score <50 (out of 100) indicating the product is unacceptable, while if the score is more than 90, the product may be superior.

### Pilot randomized controlled trial

#### Study design and participants

**Figure 3:**
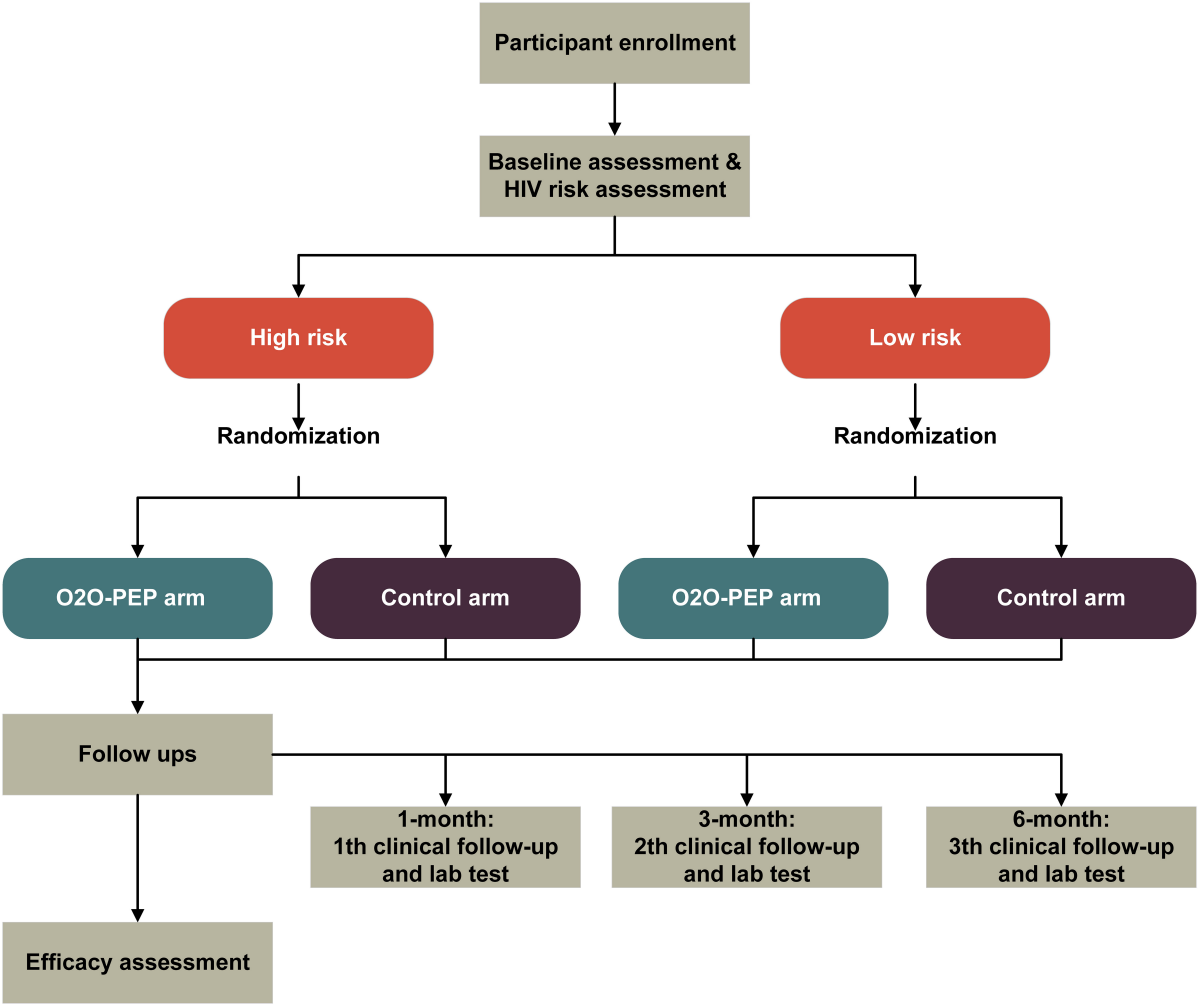
Trial profile.

The purpose of the pilot study is to assess the feasibility, acceptability, and preliminary evidence of the efficacy of the O2O-PEP model in increasing PEP uptake among MSM through a pilot two-arm stratified RCT comparing the O2O-PEP group with the standard care group (Figure 3). Participants will be recruited via online social media and offline CBOs. MSM are eligible to participate in our pilot study if they (1) are 18 years or older, (2) are assigned male at birth, (3) ever had anal intercourse with a man, 3) have HIV-uninfected by a laboratory rapid antibody test, (4) are going to reside in Jiangsu province and Qingdao city in the following 6 months after recruitment, (5) own a smartphone with Android-based operating system, (6) can access the WeChat app during the study period, and (7) are able and willing to provide electronic informed consent. Exclusion criteria include (1) self-reporting concomitant use of HIV PrEP at study entry, (2) creatinine clearance <30 ml/min, (3) known co-infection with chronic hepatitis B at enrollment, (4) participating in another research intervention related to PEP, (5) mental health disorders that may compromise adherence or safety, such as memory loss, cognition impairment, or communication disorders (assessed by an experienced clinical psychologist who is not involved in the study), and (6) presence of acute HIV infection symptoms [assessed by the Amsterdam score (31)].

#### Study settings

A convenience sample will be recruited in Jiangsu and Qingdao, China, via two local MSM-related CBOs. The two CBOs are run by non-medical community staff trained to perform rapid HIV testing and consultation, PEP and PrEP counseling, and psychological support for HIV-negative and positive clients. Both CBOs have rich experience in HIV research and management. Offline PEP prescription will be conducted at Suzhou Fifth People's Hospital (Jiangsu) and Qingdao Sixth People's Hospital (Qingdao, China), both of which are among the designated PEP treatment hospitals under the national PEP uptake guideline (32).

#### Randomization

Simple randomization, stratified by baseline HIV risk assessment (low risk vs. high risk) will be performed once the baseline survey and HIV risk assessment are finished. An HIV infection risk assessment tool specifically designed for Chinese MSM was used in our study for HIV risk assessment (33). Variables included in the tool were the number of homosexual partners, HIV-positive homosexual partners, commercial male sexual behaviors, unprotected anal intercourse with men, diagnosis of sexually transmitted diseases, sex role during anal sex with a man, recreational drug usage, and group sex with men. All variables were reported for the previous 6 months. The total score in the tool ranges from 0 to 15, with scores of 5 and above being at high risk of HIV infection. The HIV infection risk assessment tool has been externally validated in different Chinses MSM groups and showed good discrimination performance for HIV infection risk (34, 35). Participants are finally assigned to the O2O-PEP or the control group in a 1:1 ratio with a computerized randomization algorithm by SAS 9.4. The computer-generated sequence will be generated by an independent statistician who is not involved in this study. After randomization, the participant will be automatically guided to the web link associated with their allocation. Study researchers and participants are both blinded to the allocation sequence.

#### Procedures

##### Participant enrollment

To recruit participants, we will post a clickable study advertisement through the official online WeChat accounts of the two local CBOs. The advertisement includes a brief introduction about the study and a QR code, which can be scanned by the participant for study entry. Interested MSM who scan the QR code will be directed to a simplified Chinese web-based eligibility screener at the “Wen Juan Xing” web portal to determine their age, gender, sexual behaviors, HIV status, state of residence, and availability of Android operation system-based smartphone, and then directed to the electronic consent form, the baseline survey, and HIV risk assessment. All participants who meet eligibility criteria and complete the baseline survey and risk assessment will be assigned to two groups based on stratified randomization; the O2O-PEP group and the control group. In addition, participants will also be recruited in communities by the two CBO working staff who will proactively introduce this study to potential participants.

##### The intervention group (the O2O-PEP mini-app)

The O2O-PEP mini-app is the primary user-oriented platform for the intervention group. Following randomization, intervention group participants are directed to the gamification-based PEP education package, with a link to make online bookings anonymously available on the screen whenever participants want to click during the gamification procedure. After successful online booking, clients present their PEP prescription requisition e-ticket to the clinical doctor in the study-selected hospital, the clinical doctor will start the PEP regimen according to the Chinese national guideline, and the nurse in the same department will conduct essential PEP counseling when requested by participants. Participants will not be permitted to share the study advertisement with others. To restrict access to the min-app to intervention group participants only, those randomized into the intervention group will be provided a single-use verification code which will be entered to gain access to the intervention. Participants will have unlimited access to the mini-app after randomization until the end of the study.

##### The control group (routine PEP-related care)

Participants in the control group would not be directed to the O2O-PEP mini-app after randomization, but will receive HIV- and PEP-related services following the current practices in health care services, including electronic HIV prevention materials, referrals to local PEP services, and a standard procedure to access PEP through the study hospital.

##### Follow-ups

Follow-ups will be conducted at 1, 3, and 6- months after randomization. In each follow-up, participants are invited to complete a survey relating to social support, HIV health literacy, HIV risk perception, sexual and HIV risk behaviors, mental health, and PEP uptake. Each participant will be compensated 50 RMB (the currency of China), or ~$ 8. After 1 week, participants who do not respond to the investigation will be sent a reminder offering them one additional week for completion.

##### Sample size

Our previous study found that ~5% of MSM self-reported using PEP before the survey (36). A sample of ~400 participants (200 per group) would achieve 90% power to detect a moderate effect corresponding to a difference of at least 9% points (equivalent to a relative risk of 2.8 assuming a 5% prevalence of PEP usage in the control group) in PEP uptake between the intervention and control groups using a two-sided test at a 5% significance level and assuming a 10% attribution rate after 6 months. According to Cocks et al. (37) the sample size of a pilot trial should be at least 9% of that of the main planned trial. Hence, we plan to recruit 50 participants, 25 per group, to assess preliminary evidence for the further main study.

##### Outcomes and covariates of the study

Primary and secondary outcomes, covariates, and specific survey time points for each variable in the pilot RCT are shown in Table 1.

**Table 1:** Data collection and survey time points.

**Variables**	**Description/scale**	**Follow-ups (months)**			
		**Baseline**	**1**	**3**	**6**
**Primary outcomes**					
Acceptability	System usability scale (30)		√		√
Feasibility	Exposure time to the mini-app		√		√
PEP initiation	PEP initiation during study time	√	√	√	√
**Secondary outcomes**					
PEP knowledge	PEP Knowledge Scale (38)	√	√	√	√
Depression	PHQ-9 (39)	√	√	√	√
Anxiety	The Zung Self-rating anxiety scale (40)	√	√	√	√
Levesque model constructs	PEP awareness, intention to use PEP, get PEP whenever you want, PEP affordability, PEP engagement	√	√	√	√
**Covariates**					
Demographics	Age, gender, marital status, local households, personal monthly income, ethnicity, education, insurance status, employment, and sexual orientation	√			
Social support	Whether received help from any of 10 sources of social support	√	√	√	√
HIV health literacy	An eight-item index (41)	√	√	√	√
HIV infection risk perception	PHRS (42)	√	√	√	√
Sexual behaviors	Unprotected anal intercourse	√	√	√	√
MSM related stigma	The homosexual stigma scale (43)	√	√	√	√
**Laboratory testing**					
HIV Ag/Ab testing	HIV-1/2	√	√	√	√
Syphilis	Syphilis serology	√	√		√
Gonorrhea/ Chlamydia	first-catch urine, oral, urethral, and rectal mucosal secretions	√	√		
HBV	HBsAg, anti-HBs, anti-HBc	√			√
HCV	HCV antibody test	√			√
Serum creatinine	Kidney function test	√	√		
ALT, AST	Liver function test	√	√		

The primary efficacy outcome is PEP initiation. PEP initiation data will be collected through the clinical records in the study hospitals, which is a binary variable (1 = PEP initiation during the study period, otherwise 0). The acceptability outcome will be measured by the System Usability Scale (SUS) (30). For feasibility, we will assess the cumulative exposure time to the mini-app, as well as time spent on specific components of the mini-app, which will be automatically collected by participant application log files.

Secondary outcomes include (1) PEP knowledge, evaluated with an 11-item scale developed by Li et al. (39) for Chinese MSM, and the Cronbach's Alpha was 0.997 (38). (2) Depression. The Patient Health Questionnaire (PHQ-9) will be used to measure the severity of depression symptoms, which scores each item as “0” (not at all) to “3” (nearly every day). The Chinese version of PHQ-9 has been previously well-validated in people living with HIV (44). (3) Anxiety. It will be assessed by the Chinese version of the Zung Self-Rating Anxiety Scale (SAS) (40). The SAS comprises 20 items, and each item was scored on a 4-point rating, increasing scores suggested an increasing severity of anxiety symptoms. Previous studies have confirmed the reliability and validity of the SAS (Cronbach's alpha = 0.85) in the Chinese population (45, 46). (4) Finally, we will assess the Levesque model constructs including the ability to perceive, ability to seek, ability to reach, ability to pay, and ability to engage. The ability to perceive will be assessed by PEP awareness, by asking participants to report whether they have heard of PEP, with a response of “Yes” or “No.” The ability to seek will be assessed by the intention to use PEP, with the question “how likely are you to use PEP when you are at risk in the next 6 months?” with a 6-point Likert response from 1 = very unlikely to 6 = extremely likely. The ability to reach will be assessed by the question “Can you get PEP when you want to use it?” with a response of “Yes” or “No.” The ability to pay will be asked “Are you able to afford PEP when you have to pay for it out of your pocket?” with a response of “Yes” or “No.” The ability to engage will be assessed by the question “Are you able to comply with PEP usage rules?” with a response of “Yes” or “No.”

Covariate variables include demographics, social support, HIV health literacy, HIV infection risk perception, sexual behaviors, and MSM-related stigma. As for social support, participants will report on whether they receive help from any of ten sources with a 5-point rating (1 = extremely harmful to 5 = extremely helpful): primary and other sexual partners; friends; siblings; parents; medical, religious, mental health, and social service professionals; and community organizations (47). HIV health literacy will be measured by an index of eight items relating to various HIV prevention and treatment practices, responses for each item are coded as correct or incorrect, and all the corrected responses are summed to determine the total score (41). The HIV health literacy index has been used for HIV-negative MSM and is positively associated with PrEP awareness (41). HIV infection risk perception will be evaluated by the 8-item Perceived Risk of HIV infection Scale (PRHS) (42). The PRHS demonstrated good reliability and concurrent criterion-related validity, with Cronbach's coefficient of 0.79 in a Chinese college student study (48). MSM-related stigma will be assessed with the Neilands' homosexual stigma scale (43), consisting of perceived stigma and enacted stigma. This scale consists of 10 items, each scoring from “0 = never” to “3 = many times.” The scale has been translated into Chinese and the Cronbach's alpha value was 0.76 (49).

To identify potential conditions that would affect PEP usage, laboratory testing will be conducted following the national guideline. These include free and voluntary HIV rapid testing (HIV-1/antibody test), HBV test (hepatitis B surface antigen [HBsAg], hepatitis B surface antibody [anti-HBs], and hepatitis B core antibody [anti-HBc]), HCV antibody test, blood Syphilis test, Nucleic Acid Amplification Test for Gonorrhea and Chlamydia (by testing first-catch urine, oral, urethral and rectal mucosal secretions), serum creatinine, and liver function test (Alanine transaminase, Aspartate transaminase).

##### Statistical analysis plan

All statistical analyses will be conducted using SAS software (version 9.4, SAS Institute, Inc. USA), with a two-sided *p*-value of <0.05 considered statistically significant. The primary analysis will be performed by intention-to-treat analysis. Descriptive statistical analysis will be first carried out to report baseline demographics, outcome variables, and other covariates at different follow-up time points, and then compared with Chi-square/Fisher exact probability tests (for categorical variables) or *t*-test/Mann Whitney U test (for continuous variables) between those who finish follow-ups and those who do not and between the mini-app group and the control group.

The preliminary efficacy of the min-app intervention to increase PEP initiation (any PEP initiation over follow-ups) will be evaluated using an unadjusted risk ratio. As for model acceptability, point estimates for a SUS score of ≥50 will be considered the minimum criteria for acceptability. Descriptive analysis will be used to evaluate model usability, including cumulative time to the mini-app, and time spent on specific components of the mini-app. Multiple imputations will replace missing data, and a sensitivity analysis will be performed to compare the multiple imputation analysis with the full case analysis.

## Discussion

The HIV epidemic among MSM is spreading worldwide (50). Thus, promoting the O2O model application in scaling up biomedical HIV prevention interventions (e.g., PrEP and PEP) is essential. Our study aims to design the O2O-PEP model and evaluate its efficacy in promoting PEP uptake with a stratified RCT design among Chinese MSM. We propose that this O2O-PEP model will also provide opportunities for further HIV interventions (e.g., the transformation from PEP to PrEP).

Our model consists of three innovative components. First, the gamification-based PEP education packages provide a more entertaining format in the context of behavioral education, which has been adopted in other HIV interventions, such as HIV testing and shame reduction in MSM (28, 51). Second, the online HIV risk assessment tools can improve individual risk awareness and subsequent PEP-seeking actions. Third, the online booking system promotes PEP service accessibility. Furthermore, as PEP must be started within 72 h after a potential exposure to HIV, every effort should be made to provide timely PEP as soon as possible after suspicious exposure. The potential of the O2O model to deliver prompt response and dramatically reduce the linkage time makes it an ideal PEP service delivery solution.

There may be several potential challenges to the success of our study. First, the proportion of MSM being transferred from online to offline PEP prescription may be low, as PEP is relatively expensive and uncovered by Medicare in China. We try to use program funds to subsidize part of drug and laboratory test costs; second, compared with modern industrial games, the gamification-based design in our study is slow to update, which may cause study participants to lose interest. To keep participants engaged in the study, we will build in frequent releases of improved gamification content. Third, the sample size we used for primary efficacy evaluation was relatively small, and further large sample implementation studies are needed.

## Data availability statement

The original contributions presented in the study are included in the article; further inquiries can contact the corresponding author.

## Ethics statement

This study was approved by the medical institutional review board (IRB) of Binzhou Medical University (IRB No. 2021-007). All participants will sign an electronic informed consent form before enrolling in the study, which will also authorize access to their clinical records in the study hospitals. Each participant will be compensated 50 RMB each for completion of the baseline, 1, 3, and 6-month follow-ups. If the intervention proves to be effective, access to the invention is guaranteed for all high-risk MSMs even after the study has been concluded. We would also share the implementation challenges and successes in future research by reporting on international conferences and peer-reviewed articles.

## Author contributions

QL: conceptualization. QL and YL: methodology. QL, TL, and TC: writing. All authors contributed to the article and approved the final submission.

## Funding

This study was funded by the National Natural Science Foundation of China (72104033).

## Conflict of interest

The authors declare that the research was conducted in the absence of any commercial or financial relationships that could be construed as a potential conflict of interest.

## Publisher's note

All claims expressed in this article are solely those of the authors and do not necessarily represent those of their affiliated organizations, or those of the publisher, the editors and the reviewers. Any product that may be evaluated in this article, or claim that may be made by its manufacturer, is not guaranteed or endorsed by the publisher.

## References

[1] Beyrer C Baral SD van Griensven F Goodreau SM Chariyalertsak S Wirtz AL . Global epidemiology of HIV infection in men who have sex with men. Lancet. (2012) 380:367–77. 10.1016/S0140-6736(12)60821-6 22819660 PMC3805037

[2] Chow EP Wilson DP Zhang J Jing J Zhang L. Human immunodeficiency virus prevalence is increasing among men who have sex with men in China: findings from a review and meta-analysis. Sex Transm Dis. (2011) 38:845–57. 10.1097/OLQ.0b013e31821a4f43 21844741

[3] Tang S Tang W Meyers K Chan P Chen Z Tucker JD . epidemiology and responses among men who have sex with men and transgender individuals in China: a scoping review. BMC Infect Dis. (2016) 16:588. 10.1186/s12879-016-1904-5 27765021 PMC5073436

[4] Yin Y Liu Y Zhu J Hong X Yuan R Fu G . The prevalence, temporal trends, and geographical distribution of HIV-1 subtypes among men who have sex with men in China: A systematic review and meta-analysis. Epidemiol Infect. (2019) 147:e83. 10.1017/S0950268818003400 30869019 PMC6518548

[5] Xu JJ Huang XJ Liu XC Wang LM Chen YK Wang H . Consensus statement on human immunodeficiency virus pre-exposure prophylaxis in China. Chin Med J (Engl). (2020) 133:2840–6. 10.1097/CM9.0000000000001181 33273333 PMC10631579

[6] National Center for AIDS/STD Control and Prevention China CDC. Technical guidelines for HIV nonoccupational Post-Exposure Prophylaxis 2020 online at: https://ncaids.chinacdc.cn/tzgg_10268/202011/W020201116802422550750.pdf. (accessed June 20, 2022).

[7] Cardo DM Culver DH Ciesielski CA Srivastava PU Marcus R Abiteboul D . A case-control study of HIV seroconversion in health care workers after percutaneous exposure. Centers for disease control and prevention needlestick surveillance group. N Eng J Med. (1997) 337:1485–90. 10.1056/NEJM199711203372101 9366579

[8] Irvine C Egan KJ Shubber Z Van Rompay KK Beanland RL Ford N. Efficacy of HIV postexposure prophylaxis: systematic review and meta-analysis of nonhuman primate studies. Clin Infect Dis. (2015) 60 Suppl 3:S165–9. 10.1093/cid/civ069 25972498

[9] Canadian guideline on HIV pre-exposure prophylaxis and nonoccupational postexposure prophylaxis. CMAJ. (2018) 190:E782. 10.1503/cmaj.180718 29941442 PMC6019346

[10] Antiretroviral postexposure prophylaxis A or other nonoccupational exposure to HIV - United States 2016. MMWR Morb Mortal Wkly Rep. (2016) 65:458. 10.15585/mmwr.mm6517a5 27149423

[11] Cresswell F Asanati K Bhagani S Boffito M Delpech V Ellis J . UK guideline for the use of HIV post-exposure prophylaxis 2021. HIV Med. (2022) 23:494–545. 10.1111/hiv.13208 35166004

[12] Merchant RC Mayer KH Browning CA. Development of guidelines on nonoccupational HIV postexposure prophylaxis for the state of Rhode Island. Public health report. (2004) 119:136–40. 10.1177/003335490411900205 15192899 PMC1497611

[13] Wang Z Yuan T Fan S Qian HZ Li P Zhan Y . HIV Non-occupational postexposure prophylaxis among men who have sex with men: a systematic review and meta-analysis of global data. AIDS Patient Care STDS. (2020) 34:193–204. 10.1089/apc.2019.0313 32396477

[14] Rodríguez AE Castel AD Parish CL Willis S Feaster DJ Kharfen M . HIV medical providers' perceptions of the use of antiretroviral therapy as nonoccupational postexposure prophylaxis in 2 major metropolitan areas. J Acquir Immune Defic Syndr. (2013) 64 Suppl 1:S68–79. 10.1097/QAI.0b013e3182a901a2 24126450 PMC3845443

[15] Sayer C Fisher M Nixon E Nambiar K Richardson D Perry N . Will I? Won't I? Why do men who have sex with men present for post-exposure prophylaxis for sexual exposures? Sex Transm Infect. (2009) 85:206–11. 10.1136/sti.2008.033662 19074929

[16] Schechter M. do Lago RF Mendelsohn AB Moreira RI Moulton LH Harrison LH. Behavioral impact, acceptability, and HIV incidence among homosexual men with access to postexposure chemoprophylaxis for HIV. J Acq Imm Def Synd. (2004) 35:519–25. 10.1097/00126334-200404150-00010 15021317

[17] Oldenburg CE Perez-Brumer AG Hatzenbuehler ML Krakower D Novak DS Mimiaga MJ . State-level structural sexual stigma and HIV prevention in a national online sample of HIV-uninfected MSM in the United States. AID. (2015) 29:837–45. 10.1097/QAD.0000000000000622 25730508 PMC4439297

[18] Anand T Nitpolprasert C Trachunthong D Kerr SJ Janyam S Linjongrat D . A novel Online-to-Offline (O2O) model for pre-exposure prophylaxis and HIV testing scale up. J Int AIDS Soc. (2017) 20:21326. 10.7448/IAS.20.1.21326 28362062 PMC5467616

[19] Polilli E Sozio F Di Stefano P Sciacca A Ursini T Paoloni M . Web-based HIV testing in Abruzzo, Italy: analysis of 15-month activity results. AIDS Patient Care STDS. (2016) 30:471–5. 10.1089/apc.2016.0082 27749107

[20] Wang X Xu J Wu Z A. Pilot program of pre-exposure and post-exposure prophylaxis promotion among men who have sex with men - 7 study sites, China, 2018-2019. China CDC Weekly. (2020) 2:917–9. 10.46234/ccdcw2020.250 34594800 PMC8422361

[21] Levesque JF Harris MF Russell G. Patient-centred access to health care: conceptualizing access at the interface of health systems and populations. Int J Equity Health. (2013) 12:18. 10.1186/1475-9276-12-18 23496984 PMC3610159

[22] Suurmond J Rosenmöller DL El Mesbahi H Lamkaddem M Essink-Bot ML. Barriers in access to home care services among ethnic minority and Dutch elderly–a qualitative study. Int J Nurs Stud. (2016) 54:23–35. 10.1016/j.ijnurstu.2015.02.014 25776734

[23] Anand T Nitpolprasert C Phanuphak N. Online-to-offline models in HIV service delivery. Curr Opin HIV AIDS. (2017) 12:447–57. 10.1097/COH.0000000000000403 28682799 PMC5642119

[24] Guo Y Hong YA Qiao J Xu Z Zhang H Zeng C . Run4Love, a mHealth (WeChat-based) intervention to improve mental health of people living with HIV: a randomized controlled trial protocol. BMC Public Health. (2018) 18:793. 10.1186/s12889-018-5693-1 29940921 PMC6019517

[25] Jiang Y Liu F Guo J Sun P Chen Z Li J . Evaluating an intervention program using wechat for patients with chronic obstructive pulmonary disease: randomized controlled trial. J Med Internet Res. (2020) 22:e17089. 10.2196/17089 32314971 PMC7201319

[26] Plotzky C Lindwedel U Sorber M Loessl B König P Kunze C . Virtual reality simulations in nurse education: a systematic mapping review. Nurse Educ Today. (2021) 101:104868. 10.1016/j.nedt.2021.104868 33798987

[27] Ricci S Calandrino A Borgonovo G Chirico M Casadio M. Viewpoint: virtual and augmented reality in basic and advanced life support training. JMIR Serious Games. (2022) 10:e28595. 10.2196/28595 35319477 PMC8987970

[28] Christensen JL Miller LC Appleby PR Corsbie-Massay C Godoy CG Marsella SC . Reducing shame in a game that predicts HIV risk reduction for young adult MSM: a randomized trial delivered nationally over the Web. J Int AIDS Soc. (2013) 16(3 Suppl 2):18716. 10.7448/IAS.16.3.18716 24242264 PMC3833191

[29] Muessig KE Knudtson KA Soni K Larsen MA Traum D Dong W . I didn't tell you sooner because i didn't know how to handle it myself” developing a virtual reality program to support hiv-status disclosure decisions. Dig Cult Edu. (2018) 10:22–48. 30123342 PMC6097708

[30] Bangor A Kortum P Miller J. The system usability scale (SUS): an emprical evaluation. Int J Hum Comput Interact. (2008) 24:574–94. 10.1080/10447310802205776

[31] Dijkstra M de Bree GJ Stolte IG Davidovich U Sanders EJ Prins M . Development and validation of a risk score to assist screening for acute HIV-1 infection among men who have sex with men. BMC Infect Dis. (2017) 17:425. 10.1186/s12879-017-2805-y 28615005 PMC5471739

[32] National Center for AIDS/STD Control and Prevention China CDC. National HIV post-exposure prophylaxis clinic information list 2021. Available online at: https://ncaids.chinacdc.cn/tzgg_10268/202111/t20211128_253055.htm (accessed June 20, 2022).

[33] Li LL Jiang Z Song WL Ding YY Xu J He N. Development of HIV infection risk assessment tool for men who have sex with men based on Delphi method. Zhonghua Liu Xing Bing Xue Za Zhi. (2017) 38:1426–30. 10.3760/cma.j.issn.0254-6450.2017.10.026 29060993

[34] Luo Q Huang X Li L Ding Y Mi G Scott SR . External validation of a prediction tool to estimate the risk of human immunodeficiency virus infection amongst men who have sex with men. Medicine. (2019) 98:e16375. 10.1097/MD.0000000000016375 31335685 PMC6708837

[35] Zheng M He J Yuan Z Zhang X Yao Y Fang X . Risk assessment and identification of HIV infection among men who have sex with men: a cross-sectional study in Southwest China. BMJ Open. (2020) 10:e039557. 10.1136/bmjopen-2020-039557 33275116 PMC7678388

[36] Luo Q Ren X Cheng X Lin R Xue J. Comparison of HIV nonoccupational postexposure prophylaxis usage between 18 to 24 year-old student men who have sex with men and non-student men who have sex with men: a cross-sectional study. China J AIDS STD. (2021) 27:1389–93. 10.13419/j.cnki.aids.2021.12.13

[37] Cocks K Torgerson DJ. Sample size calculations for pilot randomized trials: a confidence interval approach. J Clin Epidemiol. (2013) 66:197–201. 10.1016/j.jclinepi.2012.09.002 23195919

[38] Li H Wei R Piqueiras E Chow EPF Jiao K Lewis T . HIV non-occupational postexposure prophylaxis (nPEP) usage among five key populations in China. Sex Transm Infect. (2021) 97:411–3. 10.1136/sextrans-2020-054791 33397800

[39] Kroenke K Spitzer RL Williams JB. The PHQ-9: validity of a brief depression severity measure. J Gen Intern Med. (2001) 16:606–13. 10.1046/j.1525-1497.2001.016009606.x 11556941 PMC1495268

[40] Zung WW A. rating instrument for anxiety disorders. Psychosomatics. (1971) 12:371–9. 10.1016/S0033-3182(71)71479-0 5172928

[41] Schuyler A Alidina Z Dolcini MM Harper G Fortenberry JD Singh R . Pre-exposure prophylaxis (PrEP) dissemination: adapting diffusion theory to examine PrEP adoption. AIDS Behav. (2021) 25:3145–58. 10.1007/s10461-021-03345-2 34152531 PMC11298242

[42] Napper LE Fisher DG Reynolds GL. Development of the perceived risk of HIV scale. AIDS Behav. (2012) 16:1075–83. 10.1007/s10461-011-0003-2 21785873 PMC3338003

[43] Neilands TB Steward WT Choi KH. Assessment of stigma towards homosexuality in China: a study of men who have sex with men. Arch Sex Behav. (2008) 37:838–44. 10.1007/s10508-007-9305-x 18274889

[44] Niu L Qiu Y Luo D Chen X Wang M Pakenham KI . Cross-culture validation of the HIV/AIDS stress scale: the development of a revised Chinese version. PLoS ONE. (2016) 11:e0152990. 10.1371/journal.pone.0152990 27043134 PMC4820124

[45] Zhou J Yang Y Qiu X Yang X Pan H Ban B . Relationship between anxiety and burnout among Chinese physicians: a moderated mediation model. PLoS ONE. (2016) 11:e0157013. 10.1371/journal.pone.0157013 27479002 PMC4968847

[46] Xu L Ren J Cheng M Tang K Dong M Hou X . Depressive symptoms and risk factors in Chinese persons with type 2 diabetes. Arch Med Res. (2004) 35:301–7. 10.1016/j.arcmed.2004.04.006 15325504

[47] Peterson JL Coates TJ Catania JA Middleton L Hilliard B Hearst N. High-risk sexual behavior and condom use among gay and bisexual African-American men. Am J Public Health. (1992) 82:1490–4. 10.2105/AJPH.82.11.1490 1443298 PMC1694615

[48] Jin SS Bu K Chen FF Xu HF Li Y Zhao DH . Correlates of condom-use self-efficacy on the EPPM-based integrated model among Chinese college students. Biomed Environ Sci. (2017) 30:97–105. 10.3967/bes2017.013 28292347

[49] Wang N Huang B Ruan Y Amico KR Vermund SH Zheng S . Association between stigma towards HIV and MSM and intimate partner violence among newly HIV-diagnosed Chinese men who have sex with men. BMC Public Health. (2020) 20:204. 10.1186/s12889-020-8259-y 32039716 PMC7008577

[50] UNAIDS. Global HIV & AIDS statistics-Fact sheet. (2020). Available online at: https://www.unaids.org/en/resources/fact-sheet (accessed June 22, 2022).

[51] Castel AD Wilbourn B Trexler C D'Angelo LD Greenberg D. A digital gaming intervention to improve HIV testing for adolescents and young adults: protocol for development and a pilot randomized controlled trial. JMIR Res Prot. (2021) 10:e29792. 10.2196/29792 34185022 PMC8277397

